# Spectrum of Thrombotic Complications in Fatal Cases of COVID-19: Focus on Pulmonary Artery Thrombosis In Situ

**DOI:** 10.3390/v15081681

**Published:** 2023-08-02

**Authors:** Anastasiya S. Babkina, Mikhail Y. Yadgarov, Alexey V. Volkov, Artem N. Kuzovlev, Andrey V. Grechko, Arkady M. Golubev

**Affiliations:** 1 Federal Research and Clinical Center of Intensive Care Medicine and Rehabilitology, Moscow 107031, Russia; mikhail.yadgarov@mail.ru (M.Y.Y.); artem_kuzovlev@fnkcrr.ru (A.N.K.); avg-2007@ya.ru (A.V.G.); arkadygolubev@mail.ru (A.M.G.); 2 Department of Pathological Anatomy, Institute of Medicine, Peoples’ Friendship University of Russia Named after Patrice Lumumba, Moscow 117198, Russia; alex.volkoff@gmail.com

**Keywords:** COVID-19, autopsy, thrombotic complications, pulmonary artery thrombosis, pulmonary embolism, neutrophil infiltration, pneumonia, ARDS

## Abstract

COVID-19-related thrombosis affects the venous and arterial systems. Data from 156 autopsies of COVID-19 patients were retrospectively analyzed to investigate the pattern of thrombotic complications and factors associated with pulmonary artery thrombosis and thromboembolism. Thrombotic complications were observed in a significant proportion (*n* = 68, 44%), with pulmonary artery thrombosis the most frequently identified thrombotic event (42, 27%). Multivariate analysis revealed that the length of hospital stay (OR 1.1, *p* = 0.004), neutrophil infiltration in the alveolar spaces (OR 3.6, *p* = 0.002), and the absence of hyaline membranes (OR 0.1, *p* = 0.01) were associated with thrombotic complications. Neutrophil infiltration in the alveolar spaces (OR 8, *p* < 0.001) and the absence of hyaline membranes (OR 0.1, *p* = 0.003) were also independent predictors of pulmonary artery thrombosis. The association of pulmonary artery thrombosis with an absence of hyaline membranes suggests it occurs later in the course of COVID-19 infection. As neutrophil infiltration in the alveolar spaces may indicate bacterial infection, our studies suggest the consideration of bacterial infections in these critically ill patients.

## 1. Introduction

Since the World Health Organization (WHO) declared a pandemic of coronavirus disease 2019 (COVID-19), caused by severe acute respiratory syndrome coronavirus 2 (SARS-CoV-2), on 11 March 2020 until the end of June 2023, more than 6.9 million fatal cases of COVID-19 have been reported [1]. The accumulation of knowledge about the disease has contributed to improvements in treatment, which, combined with vaccination, have led to the current decline in mortality from COVID-19. However, the pathogenesis of several complications of the disease requires further investigation.

Although COVID-19 is a respiratory disease and the lung is the target organ of SARS-CoV-2, extrapulmonary complications have been reported in both clinical and pathological studies. Cardiovascular, neurological, gastrointestinal, renal, hepatic, and other complications have been described [2,3]. Since early 2020, there have been increasing reports of hypercoagulable state, especially leading to venous thromboembolism (VTE), in patients with COVID-19 [4,5,6]. Tang et al. found that abnormal coagulation parameters, especially elevated D-dimer and fibrin degradation product levels, were predictors of a fatal outcome in COVID-19 [7]. The incidence of VTE in patients with COVID-19 ranges from 0% to 8% in general wards and from 16% to 35% in intensive care units, despite appropriate prophylaxis [8]. A high rate of VTE, up to 58%, has been reported in autopsies performed on COVID-19 patients in whom VTE was not suspected before death [9].

Since the beginning of the pandemic, the focus has been set on the prevention and treatment of VTE. Over time, however, studies of in situ arterial thrombosis in COVID-19 have emerged [10,11]. COVID-19-related thrombosis has been shown to affect both the venous and arterial vasculature of various organs, manifesting as ischemic stroke, myocardial infarction, mesenteric ischemia, limb ischemia, deep vein thrombosis, or pulmonary thromboembolism [12]. Both pulmonary embolism (PE) and in situ pulmonary artery thrombosis (PAT) can be fatal despite prophylactic anticoagulation [13].

Thrombi in the pulmonary arteries without an obvious source of embolism were found in many diseases, not only in COVID-19. Chronic obstructive pulmonary disease, bronchial asthma, sickle cell anemia, emergency and elective surgery, viral pneumonia, and other conditions may be complicated by PAT without concomitant deep vein thrombosis (DVT) [14].

The pathogenesis of arterial thrombosis differs from that of venous thrombosis, which is crucial for the diagnostic strategy and treatment of patients with thrombotic complications [15].

The restriction of autopsies in most European countries at the beginning of the pandemic significantly hampered the study of the pathogenesis of fatal complications of COVID-19, including thrombotic complications. Postmortem examination is essential for understanding disease mechanisms and potential therapeutic approaches. The importance of arterial thrombosis, venous embolism, and their combinations in the structure of COVID-19 thrombotic complications can only be objectively assessed by taking into account data from postmortem examination. Autopsies are crucial to determine the underlying causes, complications, and factors associated with fatal outcomes [16,17].

Objective: to investigate the pattern of thrombotic complications in COVID-19 with fatal outcomes and to identify factors associated with pulmonary artery thrombosis and thromboembolism.

## 2. Materials and Methods

### 2.1. Study Design and Setting

A retrospective analysis of autopsy protocols of patients who died between 1 July and 1 August 2021, was performed. Cases in which COVID-19 was the underlying cause of death and the SARS-CoV-2 virus was identified antemortem were included in the study. All autopsies were performed in the pathology department of the E.O. Mukhin State Clinical Hospital of the Moscow City Health Department in accordance with the current guidelines of the Russian Ministry of Health [18] and the legislation of the Russian Federation to verify the diagnosis and clarify the cause of death. This study was conducted in accordance with the Declaration of Helsinki on research ethics, protecting the confidentiality and dignity of the patients. No patient names or other unique identifiers were used in this study. The study protocol was approved by the local ethics committee (No. 2/21/3 of 16 April 2021).

A complete autopsy was performed on all deceased patients, including dissection of the cranial, thoracic, and abdominal cavities, and lower limbs in cases of suspected pulmonary thromboembolism. The most common sources of PE were examined, including the deep veins of the lower extremities, the pelvic and renal veins, and the inferior vena cava. The description of the organs and their pathological changes were presented in the autopsy protocols. Samples of the lungs, myocardium, brain, liver, kidneys, spleen, and pancreas were collected for histologic examination in all cases. Fixation was performed in 10% neutral buffered formalin for at least 24 h. In each case, 2 lung samples were sent for microbiological examination via polymerase chain reaction (PCR) to identify the SARS-CoV-2 virus. The results of the microbiological examination of the postmortem lung tissue and the results of the histological examination of the hematoxylin and eosin-stained specimens were attached to the autopsy protocols in all cases.

### 2.2. Variables

The following clinical and demographic characteristics of non-survivors were selected for the database and subsequent analysis: sex, age, respiratory support (invasive mechanical ventilation, non-invasive ventilation) for more than 24 h, mechanical ventilation for more than 24 h, and length of hospital stay (from admission to death). Clinical data were extracted only from the clinical summary of the autopsy protocol.

Parameters indicating the extent of lung damage (unilateral/bilateral, total/subtotal/lobar lesion, presence of hemorrhagic component, infarction of lung/lung segments, pulmonary edema, signs of bacterial infection, i.e., purulent discharge from dissected bronchi or lung) and data on extrapulmonary complications (myocardial and other organ infarction) were selected. We analyzed competing/concurrent causes of death, comorbidity, and immediate causes of death.

Thrombotic complications were identified based on the autopsy findings, the results of histologic examination, and the clinical summary of the protocol. Pulmonary embolism was diagnosed when the source of thromboembolism was identified. The source of thromboembolism was determined based on autopsy findings or imaging findings reported in the clinical summary of the protocol. Pulmonary artery thrombosis was determined by the absence of the source of the thromboembolus, the adhesion of the thrombus to the arterial wall, the enlargement of blood vessels, vessels filled with thrombus, and the presence of thrombi in large and also small branches of pulmonary arteries.

Histologic data were systematically organized, and the following features were selected for analysis: intra-alveolar and interstitial edema, intra-alveolar hemorrhage, hyaline membranes on alveolar duct or sacs, fibrosis, atypical pneumocytes, desquamation, squamous metaplasia of bronchial and alveolar epithelium, engorged blood vessels, arterial and venous thrombi, and leukocyte infiltration of lungs and bronchi (lymphocyte, neutrophil). Based on the signs characterizing the stages of lung damage, we determined exudative/acute (edema, hyaline membranes, hemorrhage), organizing/subacute (fibroblast proliferation, remnants of hyaline membrane with or without organization, lymphocytic infiltration), and fibrotic (focal fibrosis) phases.

### 2.3. Statistical Analysis

The normality of data distribution was assessed using the Shapiro–Wilk test. Descriptive statistics such as mean and standard deviation (SD) were calculated for data with normal distribution. Non-normal data were described using median and interquartile range (IQR). Categorical data were presented as frequencies and percentages. The Mann–Whitney U test and Student’s *t*-test for continuous variables were used to assess group differences, depending on the normality of the data. Chi-squared test, Fisher’s exact test, and the Fisher–Freeman–Halton exact test were used for categorical baseline variables. In addition, multivariate regression analysis was performed to determine adjusted odds ratios, taking into account the effect of confounding variables. The backward stepwise Wald method was used for variable selection. All statistical analyses were performed with IBM SPSS Statistics for Windows, version 27.0 (IBM Corp., Armonk, NY, USA). The significance level was set at 0.05.

## 3. Results

### 3.1. Descriptive Statistics

The clinical and demographic characteristics of patients and their postmortem diagnoses are presented in Table 1 and Supplementary Materials. The total number of eligible patients was 156. The mean length of hospital stay was 9 days (IQR 5–13). Most patients received respiratory support for more than 24 h (100 cases, 64%). Invasive mechanical ventilation for more than 24 h before death was used in 24 cases (15%). Of the 156 deaths with COVID-19 confirmed ante-mortem, the virus was identified in postmortem lung tissue in 66 cases (42%). 

Lung injury was characterized as bilateral and total in most cases. Pulmonary edema with a hemorrhagic component was observed in almost all cases (153 out of 156). In 15 cases (9.6%) there were signs of purulent pneumonia at autopsy. In cases where bacteriologic findings were attached to the autopsy protocols, the causative agents of pneumonia were *Acinetobacter* (4 cases), *Klebsiella* spp. (3 cases), *Enterobacter* (1 case), *Pseudomonas aeruginosa* (1 case), and *Proteus* spp. (2 cases).

The most common immediate cause of death was respiratory failure (125 cases, 80%). Other important causes were massive pulmonary embolism (11 cases, or 7.1% of deaths) and cardiorespiratory failure due to the combination of COVID-19 with cardiovascular comorbidity (15 cases, or 9.6% of deaths). Cerebral edema with herniation occurred in four cases and was caused by ischemic stroke in two cases, hemorrhagic stroke in one case, and the absence of acute cerebrovascular accident in one case. Heart failure was the immediate cause of death in type 2 myocardial infarction. 

The most common comorbidities were hypertension (144 cases, 92%), atherosclerosis (146 cases, 94%), chronic respiratory diseases (113 cases, 72%), and gastrointestinal and liver diseases (113 cases, 72%) (Table 1).

Thrombotic complications (68 cases, 44%) accounted for a significant proportion of COVID-19 complications. Thrombotic complications (Table 2) included thrombosis of segmental and subsegmental branches of the pulmonary artery (42 cases, 27%). Pulmonary embolism was observed in 16 cases. The source of thromboembolism was mostly lower extremity veins (14/16), but in two cases, the thrombi originated from the right atrium. In addition to the pulmonary circulation, thromboses were detected in coronary arteries (three cases, 1.9%), renal arteries (one case, 0.6%), cerebral arteries (three cases, 1.9%), aorta (three cases, 1.9%), and right atrium (four cases, 2.6%).

Some of the thrombotic complications were fatal. Thus, pulmonary thromboembolism (from lower limb veins [9 cases] or right atrium [2 cases]) caused death in 11 cases, cerebral thrombosis caused ischemic stroke and cerebral edema with herniation in 2 cases, while coronary thrombosis caused myocardial infarction in 3 cases.

Morphological changes in the lungs corresponded to those described in acute respiratory distress syndrome (ARDS) at different phases. The main morphological changes are presented in Table 3. Signs of fibrotic (119 cases, 76%) and organizing (32 cases, 21%) phases were observed in most of the non-survivors, indicating that most of the deaths occurred in the late phase of the disease. Signs of the acute phase were noted only in five patients. The predominant infiltration with lymphocytes (142 cases, 91%) corresponds to the picture of a viral infection. Approximately one-third of the patients (54 cases, 35%) had foci of extensive neutrophil infiltration filling the alveolar spaces.

Analysis of the incidence of pulmonary vascular thrombosis according to phase (Table 4) showed that thrombosis was predominantly detected in combination with signs of fibrotic and organizing phases. None of the cases corresponding to the acute phase showed arterial thrombosis in situ.

### 3.2. Predictors of Thrombotic Complications

When comparing the frequency of the studied parameters in the groups of patients with and without thrombotic complications (Table 5), we found that patients with thrombotic complications had a longer hospital stay (11 [IQR 7–16] vs. 8 [IQR 5–11], *p* = 0.002), more frequent signs of bacterial pneumonia at autopsy (12 [18%] vs. 3 [3.4%], *p* = 0.005), neutrophil infiltration in the alveolar spaces (38 [56%] vs. 16 [18%], *p* < 0.001), chronic kidney and urinary tract disease (18 [27%] vs. 11 [13%], *p* = 0. 026), neurodegenerative disease (18 [27%] vs. 5 [5.7%], *p* < 0.001), pulmonary infarction (7 [10%] vs. 0 [0%], *p* = 0.002), and renal infarction (4 [5.9%] vs. 0 [0%], *p* = 0.03). In contrast, pneumocyte desquamation (49 [72%] vs. 76 [86%], *p* = 0.026) and hyaline membranes (48 [71%] vs. 86 [98%], *p* < 0.001) were reported less frequently in patients with thrombotic complications.

Differences were observed in the immediate causes of death between patients with and without thrombotic complications (*p* < 0.001). Specifically, cardiorespiratory failure was more common (12% [eight patients] vs. 8.0% [seven patients]) in patients with thrombotic complications, and cerebral edema with herniation was slightly more prevalent (2.9% [two patients] vs. 2.3% [two patients]), due to coronary and cerebral artery thrombosis leading to infarction.

### 3.3. Regression Analysis

Multivariate analysis revealed that the independent predictors of thrombotic complications were the length of hospital stay (OR 1.1, *p* = 0.004), neutrophil infiltration in the alveolar spaces (OR 3.6, *p* = 0.002), and the absence of hyaline membranes (OR 0.1, *p* = 0.01) (Table 6).

Neutrophil infiltration in the alveolar spaces (OR 8, *p* < 0.001) and the absence of hyaline membranes (OR 0.1, *p* = 0.003) were independent predictors of pulmonary artery thrombosis in situ (Table 7).

No factors associated with pulmonary embolism (originating in the veins of the lower extremities) were identified (Table 8).

## 4. Discussion

The analysis of 156 autopsy protocols showed that thrombotic complications of COVID-19 are not limited to the pulmonary vasculature and lower extremity veins, but also include thrombosis of the right atrium, aorta, coronary arteries, renal arteries, and cerebral arteries. Thrombotic complications are often the immediate cause of death, as confirmed by our study. Since the beginning of the pandemic, most reports on thrombotic complications have focused on the problem of deep vein thrombosis and pulmonary embolism [19,20,21]. Although thrombus formation within the right atrium, right ventricle can lead to consecutive pulmonary embolism, this issue is under-reported in the scientific literature and clinical guidelines [22,23,24,25]. There are also few data on aortic thrombosis in COVID-19 and its combination with venous thrombosis [24,26,27,28,29,30]. The results of our study demonstrated that in situ pulmonary artery thrombosis is the most common thrombotic complication identified postmortem. 

### 4.1. Arterial Thrombosis In Situ in COVID-19 and Other Lung Diseases

The issue of arterial thrombosis in situ in COVID-19 became more prominent after complete postmortem studies were performed showing thrombi in pulmonary artery branches despite anticoagulant therapy, not always associated with leg vein thrombosis [13,31]. However, this phenomenon is not specific to COVID-19. Prior to the COVID-19 pandemic, numerous studies have reported evidence for the possibility of in situ thrombus formation in the pulmonary arteries in the absence of leg vein DVT. Benns et al. showed that 84.2% of patients after severe trauma with PE did not have DVT [32]. Paffrath et al. showed that 37% of patients with PE did not have DVT [33]. Based on the evaluation of patients in the post-traumatic period using duplex sonography, PE without DVT was found to be more common than PE with DVT [34]. It can be assumed that PE without DVT is a result of the embolization of the entire venous clot. However, the results of a study by Van Gent et al. suggest that, in most cases, only a portion of the clot is dislodged to form the embolus [34]. Cases of detection of thrombi in the branches of the pulmonary artery in the absence of deep vein thrombosis (DVT) cannot be explained only by defects in the methods for diagnosing thrombosis [14]. The results of the study using total-body magnetic resonance imaging scans to visualize thrombi confirmed the high possibility of thrombosis in the pulmonary artery with no thrombi in the deep veins [35]. In situ pulmonary artery thrombus formation has been described in tuberculosis [36], cancer patients [37,38], primary pulmonary hypertension [39], pulmonary aspergillosis [40], and other diseases. 

In situ PAT is an underestimated cause of circulatory disorders in the lungs. PAT is usually peripheral and is infrequently associated with signs of right heart overload [41]. The lack of a reference standard limits clinicians’ ability to diagnose in situ PAT. The diagnostic strategy should focus on the differential diagnosis between PE and PAT. A comprehensive approach, combining clinical assessment with advanced imaging techniques, including computed tomographic venography of the lower extremity and pelvic veins, computed tomographic angiography of the lungs, magnetic resonance imaging, and duplex sonography, can provide a clear image of pulmonary vessels, identification of thrombotic occlusions, and identification of the source of embolism.

The increased attention to the issue of venous thrombosis in COVID-19 has impacted the treatment strategy and preventive measures [18,42,43]. However, the ultrasound examination of the lower limbs and the prescription of low-molecular-weight heparins to all hospitalized patients with no contraindications to anticoagulant therapy did not completely solve the problem of thrombotic complications. Moreover, the ACTIV-4a study refuted the hypothesis that routine therapeutic-dose anti-coagulation benefits critically ill patients with COVID-19 [44].

Since our study was based on clinical autopsy reports, we can assume that all patients received standard prophylactic doses of low-molecular-weight heparin according to the provisional guidelines in force at the time [18,42]. However, the frequency of thrombotic complications and the prevalence of arterial thrombosis over venous thrombosis in our study confirms the need to improve methods of preventing thrombotic complications, taking into account the pathogenesis of arterial and venous thrombosis.

### 4.2. Pathogenesis of Arterial and Venous Thrombosis

The pathogenesis of venous thrombosis is characterized by clot formation under conditions of slow blood flow, which is associated with the accumulation of active coagulation factors at the site of vascular injury. Venous thrombi consist mainly of erythrocytes and large amounts of fibrin. The activation of the coagulation system is the main cause of venous thrombosis and precedes platelet activation and aggregation [15,45]. This explains why anticoagulant therapy has become the main strategy for the treatment and prevention of venous thromboembolism.

Arterial thrombus develops under conditions of rapid blood flow. The fibrin component of the thrombus increases as it spreads into the arterial lumen and becomes abundantly coated with activated platelets. A loose network of fibrin with red blood cells also appears within the thrombus in the final stage of thrombosis. Because of the important role of platelets in arterial thrombus formation, they may be a therapeutic target for the prevention of arterial thrombosis [15].

Major mechanisms involved in the development of thrombosis in COVID-19 include endothelial damage due to inflammation, cytokine-mediated immunopathological responses, lymphocyte cell death, hypoxia [46], oxidative stress, direct viral infection of endothelial cells [13,47], hypercoagulation [48], dysregulation of the renin-angiotensin-aldosterone system and the role of ACE2, and activation of von Willebrand factor [49]. The role of local pulmonary factors in the pathogenesis of in situ thrombosis was demonstrated in the study by Fletcher-Sanfeliu et al. who found an association of pulmonary artery thrombosis in situ with chronic obstructive pulmonary disease and respiratory infections, in contrast to thromboembolism originating from DVT [50]. The association of in situ thrombosis with local factors (neutrophil infiltration, hyaline membranes) was also confirmed in our study.

### 4.3. Role of Neutrophils in Thrombosis Pathogenesis

By multivariate analysis, we found that lung neutrophil infiltration was associated with thrombotic complications, especially in situ pulmonary artery thrombosis.

The role of neutrophils in pulmonary artery thrombosis was confirmed in an experimental study by Porembskaya et al. in rats with normal neutrophil counts and neutropenia. Neutropenia was associated with a significant reduction in thrombus size in the inferior vena cava and delayed the transition from immature to mature fibrin and connective tissue formation within the thrombus. Animals with neutropenia had no thrombus in the pulmonary artery, and neutrophil ablation also eliminated macroscopic signs of lung injury [51].

The results of studies of lung samples from non-survivors of COVID-19 patients, as well as clots, confirm the pathogenetic link between inflammation and thrombosis [52,53]. For example, the study of twenty arterial thrombi revealed a large number of leukocytes, predominantly neutrophils, in their composition [53].

The mechanism of neutrophil involvement in thrombosis can be explained by NETosis. Neutrophil extracellular traps (NETs) may contribute to thrombosis by enhancing endothelial activation and the release of von Willebrand factor (VWF) from Weibel-Palade bodies. NETs are very large structures and may contribute to thrombus stability similar to VWF and fibrin. In vitro, NETs have been shown to provide a framework for clots that are resistant to tissue plasminogen activator (tPA)-induced thrombolysis [54].

The role of neutrophil extracellular traps has also been confirmed in the pathogenesis of thrombotic responses in COVID-19 [55].

### 4.4. The Role of Bacterial Infection in the Pathogenesis of Thrombosis

In 15 cases included in our study, there were signs of purulent pneumonia at autopsy. More frequent signs of bacterial pneumonia at autopsy were noted in patients with thrombotic complications (12 [18%] vs. 3 [3.4%], *p* = 0.005). It should be noted that signs of purulent pneumonia at autopsy indicate advanced infection. The initial phase of bacterial pneumonia is characterized by neutrophil-mediated inflammation [56,57,58]. The infiltration of neutrophils penetrating from capillaries into virus-infected areas in the absence of any evidence of secondary bacterial infection may be observed; however, neutrophilic infiltration is never massive [59]. Foci of extensive neutrophil infiltration filling the alveolar spaces, found in more than one-third of non-survivors, may indicate bacterial co-/super- infection not diagnosed clinically and on macroscopic postmortem examination.

Elabbadi et al. found a high prevalence of early bacterial co-infection in patients with severe COVID-19, with a high proportion of *Staphylococcus Aureus* [60]. The study by Bychinin et al. in patients with severe and critical COVID-19 showed a high incidence of nosocomial infection (48.8%), which negatively affected the disease outcome. In more than half of the cases, the infection was caused by resistant strains of *Acinetobacter baumannii* and *Klebsiella pneumoniae* [61].

According to the study by Ripa et al., thrombotic complications in patients with COVID-19 were associated with a high risk of secondary infections. Their etiology was similar in patients with and without thrombotic complications [62].

*Klebsiella pneumoniae* and *Streptococcus pneumoniae* have been shown to cause local coagulation activation and fibrin deposition in the lung [63]. NETosis activation has been observed in bacterial infections caused by *Klebsiella pneumoniae*, with *Pseudomonas aeruginosa* causing ventilator-associated pneumonia [64,65,66]. Considering the data from the few bacteriologic studies available to us during our study, the bacterial pathogens detected in COVID-19 may play a leading role in pulmonary artery thrombosis.

The role of bacterial infection has also been reported in lower extremity venous thrombosis. A study by Vakhitov et al. reported the presence of bacterial DNA, predominantly from the *Streptococcus mitis* group, in thrombus aspirates from surgical patients with arterial and deep venous thrombosis of the lower extremities [67].

We have previously found that bacterial pneumonia is associated with increased intensity of immunohistochemical (IHC) staining for VWF in the endothelium of pulmonary vessels [68]. 

### 4.5. Pathogenesis of Thrombosis According to Stage of Lung Injury in COVID-19

The association of thrombotic complications, especially pulmonary artery thrombosis, with the absence of hyaline membranes can be explained by the fact that most patients with thrombosis had a fibrotic stage, which corresponds to a later period of the disease, while hyaline membranes are more typical of the acute and early proliferative stages. There are a few cases corresponding to morphological changes in the acute stage, but none of them had in situ pulmonary vascular thrombosis. Therefore, the association of pulmonary artery thrombosis with an absence of hyaline membranes suggests it occurs later in the course of COVID-19 infection.

Limitations. Our study has several limitations including its retrospective design, which may introduce inherent biases and limit causal inference. Therefore, we cannot completely exclude defects in the method of searching for the sources of PE during autopsy. Autopsy reports also varied in the format of clinical resume and postmortem ancillary investigations; in some cases, data such as disease duration/date of manifestation of the disease, results of laboratory studies, instrumental studies, and postmortem bacteriology examinations of the lung were incomplete. Our study lacks clinical and laboratory data that can be used to predict the presence of thrombosis prior to death. Our research, given its cross-sectional design, does not permit the establishment of causal relationships. Additionally, the single-center setting may restrict the generalizability of our findings to other populations or healthcare settings. Future studies with larger, multicenter cohorts to validate and further investigate these associations may be worth conducting.

## 5. Conclusions

Thrombotic complications in COVID-19 represent a broad spectrum of both arterial and venous thrombosis, not limited to the pulmonary vasculature. However, pulmonary vascular thrombosis is the most common autopsy finding. According to the results of our study, segmental and subsegmental pulmonary artery thrombosis outnumbered other thrombotic complications, including pulmonary embolism. The association of pulmonary artery thrombosis with an absence of hyaline membranes suggests it occurs later in the course of COVID-19 infection. Foci of extensive neutrophil infiltration filling the alveolar spaces, found in more than one-third of non-survivors, is associated with pulmonary arterial thrombosis in situ. Considering that such neutrophil infiltration may indicate bacterial co-/super- infection and the proven role of some bacteria in the pathogenesis of thrombosis, more attention should be paid to the prompt diagnosis of bacterial infection in COVID-19 patients.

## Figures and Tables

**Table 1:** Characteristics of non-survivors with COVID-19 included in the study.

Parameter	Patients (*n* = 156)
Clinical and demographic characteristics	
Sex, male	68 (44%) 70.4 (SD 13.8)
Age, years	
Length of stay, days	9 (IQR 5–13)
Respiratory support >24 h	100 (64%)
Invasive mechanical ventilation >24 h	24 (15%)
PCR-confirmed COVID-19 (ante-mortem)	156 (100%)
PCR-confirmed COVID-19 (in the lungs tissue post-mortem)	66 (42%)
Competing/concomitant cause of death	33 (21%)
Complications of COVID-19	
Pulmonary edema	151 (97%)
Bilateral/unilateral lung injury	153/3
Total/subtotal/lobar lung lesion	90/58/8
Hemorrhagic component	153 (98%)
Bacterial pneumonia signs (autopsy)	15 (9.6%)
Pulmonary infarction	7 (4.5%)
Thrombotic complications	68 (44%)
Myocardial infarction	6 (3.8%)
Stroke	3 (1.9%)
Spleen infarction	2 (1.3%)
Renal infarction	4 (2.6%)
Immediate cause of death	
Respiratory failure	125 (80%)
Cardiorespiratory failure	15 (9.6%)
Massive pulmonary embolism	11 (7.1%)
Cerebral edema with herniation	4 (2.6%)
Acute heart failure	1 (0.6%)
Comorbidity	
Hypertension	144 (92%)
Atherosclerosis	146 (94%)
Coronary heart disease	26 (17%)
Obesity	54 (35%)
Diabetes mellitus	45 (29%)
Chronic respiratory disease	113 (72%)
Chronic kidney and urinary tract disease	29 (19%)
Chronic gastrointestinal diseases	113 (72%)
Chronic liver disease	91 (58%)
Neurodegenerative diseases	23 (15%)
Cerebrovascular disease	20 (13%)
Malignant neoplastic disease	17 (11%)

**Table 2:** Thrombotic complications in COVID-19.

Thrombotic Complications	Patients (*n* = 156)
Pulmonary artery embolism (source of thromboembolism identified)	16 (10%)
Deep vein thrombosis of the lower extremities	18 (12%)
Pulmonary vein thrombosis	9 (5.8%)
Pulmonary artery thrombosis	42 (27%)
Aortic thrombosis	3 (1.9%)
Right atrium thrombi	4 (2.6%)
Coronary artery thrombosis	3 (1.9%)
Renal artery thrombosis	1 (0.6%)
Cerebral artery thrombosis	3 (1.9%)

**Table 3:** Lung morphologic changes in COVID-19.

Lung Histology	Patients (*n* = 156)
Fibrosis	119 (76%)
Interstitial/alveolar edema	42 (27%)
Hyaline membranes	134 (86%)
Neutrophil infiltration in the alveolar spaces	54 (35%)
Neutrophil infiltration in the bronchi	31 (20%)
Lung lymphocytic infiltration	142 (91%)
Lymphocytic infiltration of the bronchi	29 (19%)
Vascular congestion	129 (83%)
Alveolar hemorrhage	65 (42%)
Squamous metaplasia	22 (14%)
Pneumocytes with nuclear atypia	25 (16%)
Thrombosis of arteries/arterioles	32 (21%)
Thrombosis of veins/venules	5 (3.2%)
Desquamation of pneumocytes	125 (80%)
Desquamation of bronchial epithelial cells	118 (76%)
Acute phase signs	5 (3.2%)
Organizing phase signs	32 (21%)
Fibrotic phase signs	119 (76%)

**Table 4:** Frequency of pulmonary vascular thrombosis by disease stage.

Phase	Acute, *n* = 5	Fibrotic, *n* = 119	Organizing, *n* = 32	*p* Value
Pulmonary artery thrombosis	0 (0%)	32 (27%)	10 (31%)	0.4 *
Pulmonary vein thrombosis	1 (20%)	7 (5.9%)	1 (3.1%)	0.3 *
Pulmonary embolism	1 (20%)	14 (12%)	1 (3.1%)	0.2 *

* Fisher–Freeman–Halton exact test.

**Table 5:** Frequency of the studied parameters in the groups of patients with and without thrombotic complications.

Parameter	Patients without Thrombotic Complications (*n* = 88)	Patients with Thrombotic Complications (*n* = 68)	*p* Value
Clinical and demographic characteristics			
Sex, male	37 (42%) 70 (SD 14)	31 (46%)	0.7 ^3^
Age, years		70 (SD 14)	0.9 ^1^
Length of stay, d	**8 (IQR 5–11)**	**11 (IQR 7–16)**	**0.002 ^2^**
Respiratory support >24 h	56 (64%)	44 (65%)	0.9 ^3^
Invasive mechanical ventilation >24 h	14 (16%)	10 (15%)	0.8 ^3^
PCR-confirmed COVID-19 (ante-mortem)	88 (100%)	68 (100%)	0.9 ^4^
PCR-confirmed COVID-19 (in the lungs tissue post-mortem)	38 (43%)	28 (41%)	0.9 ^3^
Competing/concomitant cause of death	11 (13%)	12 (18%)	0.4 ^3^
Complications of COVID-19			
Pulmonary edema	86 (98%)	65 (96%)	0.7 ^4^
Bilateral/unilateral lung injury	86/2	67/1	0.9 ^4^
Total/subtotal/lobar lung lesion	44/38/6	46/20/2	0.08 ^3^
Hemorrhagic component	86 (98%)	67 (99%)	0.9 ^4^
Bacterial pneumonia signs (autopsy)	**3 (3.4%)**	**12 (18%)**	**0.005 ^4^**
Pulmonary infarction	**0 (0%)**	**7 (10%)**	**0.002 ^4^**
Myocardial infarction	2 (2.3%)	4 (5.9%)	0.4 ^4^
Stroke	1 (1.1%)	2 (2.9%)	0.6 ^4^
Spleen infarction	0 (0%)	2 (2.9%)	0.2 ^4^
Renal infarction	**0 (0%)**	**4 (5.9%)**	**0.034 ^4^**
Immediate cause of death			
Respiratory failure	**78 (89%)**	**47 (69%)**	
Cardiorespiratory failure	**7 (8.0%)**	**8 (12%)**	
Massive pulmonary embolism	**0 (0%)**	**11 (16%)**	**<0.001 ^5^**
Cerebral edema with herniation	**2 (2.3%)**	**2 (2.9%)**	
Acute heart failure	**1 (1.1%)**	**0 (0%)**	
Comorbidity			
Hypertension	81 (92%)	63 (93%)	0.9 **^4^**
Atherosclerosis	82 (93%)	64 (94%)	0.9 **^4^**
Coronary heart disease	14 (16%)	12 (18%)	0.8 **^3^**
Obesity	34 (39%)	20 (29%)	0.2 **^3^**
Diabetes mellitus	28 (32%)	17 (25%)	0.4 **^3^**
Chronic respiratory disease	63 (72%)	50 (74%)	0.8 **^3^**
Chronic kidney and urinary tract disease	**11 (13%)**	**18 (27%)**	**0.026 ^3^**
Chronic gastrointestinal diseases	61 (70%)	52 (77%)	0.3 **^3^**
Chronic liver disease	56 (64%)	35 (52%)	0.13 **^3^**
Neurodegenerative diseases	**5 (5.7%)**	**18 (27%)**	**<0.001 ^4^**
Cerebrovascular disease	8 (9.1%)	12 (18%)	0.15 **^4^**
Malignant neoplastic disease	10 (11%)	7 (10%)	0.9 **^4^**
Lung morphologic changes			
Fibrosis	64 (73%)	55 (81%)	0.2 **^3^**
Interstitial/alveolar edema	28 (32%)	14 (21%)	0.1 **^3^**
Hyaline membranes	**86 (98%)**	**48 (71%)**	**<0.001 ^3^**
Neutrophil infiltration in the alveolar spaces	**16 (18%)**	**38 (56%)**	**<0.001 ^3^**
Neutrophil infiltration of the bronchi	13 (15%)	18 (27%)	0.069 **^3^**
Lung lymphocytic infiltration	78 (89%)	64 (94%)	0.2 **^3^**
Lymphocytic infiltration in the bronchi	20 (23%)	9 (13%)	0.13 **^3^**
Vascular congestion	72 (82%)	57 (84%)	0.7 **^3^**
Alveolar hemorrhage	32 (36%)	33 (49%)	0.13 **^3^**
Squamous metaplasia	9 (10%)	13 (19%)	0.16 ^4^
Pneumocytes with nuclear atypia	11 (13%)	14 (21%)	0.2 **^3^**
Thrombosis of arteries/arterioles	**0 (0%)**	**32 (47%)**	**<0.001 ^4^**
Thrombosis of veins/venules	**0 (0%)**	**5 (7.4%)**	**0.014 ^4^**
Desquamation of pneumocytes	**76 (86%)**	**49 (72%)**	**0.026 ^3^**
Desquamation of bronchial epithelial cells	71 (81%)	47 (69%)	0.095 **^3^**
Acute phase signs	3 (3.4%)	2 (2.9%)	0.5 **^3^**
Organizing phase signs	21 (23.9%)	11 (16%)	
Fibrotic phase signs	64 (72.7%)	55 (81%)	

The statistically significant results are highlighted in bold. ^1^ Student’s *t*-test; ^2^ Mann–Whitney U test, ^3^ Chi-square test; ^4^ Fisher’s exact test; ^5^ Fisher–Freeman–Halton exact test.

**Table 6:** Univariable and multivariable analysis for prediction of thrombotic complications.

Parameter	Univariable Analysis			Multivariable Analysis		
	OR	95% CI	*p* Value	OR	95% CI	*p* Value
Length of stay, days	**1.11**	**1.04–1.18**	**0.001**	**1.11**	**1.03–1.19**	**0.004**
Bacterial pneumonia signs (autopsy)	6.07	1.64–22.48	0.007	NS		0.9
Chronic kidney and urinary tract disease	2.52	1.1–5.78	0.029	NS		0.3
Neurodegenerative diseases	5.98	2.09–17.1	<0.001	NS		0.2
Hyaline membranes	**0.06**	**0.01–0.25**	**<0.001**	**0.12**	**0.03–0.60**	**0.01**
Neutrophil infiltration in the alveolar spaces	**5.7**	**2.77–11.74**	**<0.001**	**3.61**	**1.60–8.18**	**0.002**
Desquamation of pneumocytes	0.41	0.18–0.91	0.029	NS		0.8

The statistically significant results are highlighted in bold. NS, non-significant.

**Table 7:** Univariable and multivariable analysis for prediction of pulmonary artery thrombosis.

Parameter	Univariable Analysis			Multivariable Analysis		
	OR	95% CI	*p* Value	OR	95% CI	*p* Value
Length of stay, days	1.09	1.02–1.15	0.008	NS		0.06
Bacterial pneumonia signs (autopsy)	9.76	2.91–32.78	<0.001	NS		0.7
Chronic kidney and urinary tract disease	2.75	1.18–6.37	0.019	NS		0.2
Neurodegenerative diseases	5.83	2.29–14.87	<0.001	NS		0.5
Hyaline membranes	**0.05**	**0.02–0.16**	**<0.001**	**0.14**	**0.04–0.52**	**0.003**
Neutrophil infiltration in the alveolar spaces	**13.38**	**5.73–31.27**	**<0.001**	**7.95**	**3.12–20.25**	**<0.001**
Desquamation of pneumocytes	0.21	0.09–0.47	<0.001	NS		0.4

The statistically significant results are highlighted in bold. NS, non-significant.

**Table 8:** Univariable analysis for prediction of pulmonary embolism *.

Parameter	Univariable Analysis		
	OR	95% CI	*p* Value
Length of stay, days	1.06	0.97–1.15	0.07
Bacterial pneumonia signs (autopsy)	1.40	0.29–6.83	0.7
Chronic kidney and urinary tract disease	0.73	0.15–3.47	0.2
Neurodegenerative diseases	NA		0.3
Hyaline membranes	NA		0.9
Neutrophil infiltration in the alveolar spaces	0.51	0.14–1.9	0.8
Desquamation of pneumocytes	1.57	0.33–7.4	0.6

NA, not applicable. Two patients with pulmonary embolism (originating in the right atrium) were excluded.

## Data Availability

The data presented in this study are available in supplementary material. No patient names or other unique identifiers were used in this study.

## Supplementary Materials

The following supporting information can be downloaded at: https://www.mdpi.com/article/10.3390/v15081681/s1, Table S1: Data on the non-survivors with COVID-19 included in the studies.
